# Do perilunate dislocations and fracture-dislocations result in different radiological outcomes following wrist alignment reconstruction? A single-center retrospective study including 51 patients with perilunate injuries

**DOI:** 10.1007/s00402-024-05744-1

**Published:** 2025-01-03

**Authors:** Katarzyna Rachunek-Medved, Fabian Medved, Wojciech Besz, Claudius Illg, Henrik Lauer, Sabrina Krauß, Adrien Daigeler, Johannes Tobias Thiel

**Affiliations:** Department of Hand, Plastic, Reconstructive and Burn Surgery, BG Unfallklinik Tuebingen, Eberhard Karls University Tuebingen, Tübingen, Germany

**Keywords:** Perilunate luxation, Perilunate dislocations, Perilunate fracture dislocations, Wrist surgery, Wrist arthrosis

## Abstract

**Introduction:**

Perilunate dislocations (PLD) and perilunate fracture-dislocations (PLFD) are high-energy wrist injuries often linked to significant post-traumatic osteoarthritis. This study aims to determine whether PLD and PLFD yield different radiological outcomes following surgical treatment while identifying prognostic factors for worse outcomes.

**Materials and methods:**

We retrospectively analyzed 51 patients treated for perilunate injuries between 2000 and 2022. Radiographic evaluation included postoperative carpal alignment, scapholunate distance, ulnar translocation, and postoperative arthrosis according to the Kellgren-Lawrence scale. Logistic regression models were used in the study. The analyzed explanatory variables included: type of injury (PLFD/PLD), Mayfield classification, capsulodesis, repair of intercarpal- and extrinsic ligaments, and number of wrist transfixations. The significance level was set at *p* ≤ 0.05. The calculations were performed with R (version 4.3.2).

**Results:**

Among 51 patients, the mean follow-up was 4.33 years (1-22.13), and the mean age was 37.76 years. PLFD accounted for 55% of cases. Patients in the PLD group were older at the time of injury (*p* = 0.0031) compared to PLFD. Older patients presented also with higher stages of perilunate instability (*p* = 0.0061). Midcarpal arthrosis was the most common site of wrist degeneration (58.8%). Ordinal logistic regression indicated that PLFD was associated with a lower risk of midcarpal arthrosis (OR = 0.293, *p* = 0.04), while a higher number of wrist transfixations increased the risk of advanced arthrosis (OR = 2.427, *p* = 0.02), The logistic regression model detected a positive effect of the number of wrist transfixations on lunate fovea arthrosis (*p* = 0.048). The number of wrist transfixations did not correlate with the number of fractures (*p* = 0.06), Mayfield classification (*p* = 0.16), or intraoperative reduction outcome (*p* = 0.6).

**Conclusion:**

PLD and a greater number of wrist transfixations were associated with a higher risk of wrist arthrosis. Limiting wrist pinning to essential procedures may help prevent additional iatrogenic chondral lesions.

## Introduction

Perilunate dislocations (PLD) and perilunate fracture dislocations (PLFD) represent high-energy traumatic injuries with the potential to cause significant long-term morbidity and functional impairment. A PLD is defined as the displacement of the carpal bones surrounding the lunate, without the presence of associated fractures [1–4]. In contrast, PLFD involves dislocation of the carpal bones adjacent to the lunate, in conjunction with fractures of one or more carpal bones. The bones most commonly affected in cases of PLFD are the scaphoid, capitate and triquetrum [1–4].

Perilunate injuries account for less than 10% of all wrist traumas and are most frequently observed (in 90% of cases) in young men between the ages of 25 and 40 years [1, 5, 6]. The most common complication following perilunate luxations is post-traumatic osteoarthritis of the wrist [2–4, 7, 8]. A meta-analysis conducted by Lee et al. revealed that osteoarthritis developed in approximately one-third of patients with this injury after a minimum follow-up of 12 months [7]. Similarly, Liechti et al. observed that the incidence of osteoarthritis increased significantly with extended follow-up periods [4]. Notably, despite the presence of osteoarthritis, there was no apparent correlation with diminished wrist function or patient dissatisfaction. In contrast to PLDs, PFLDs appear to be less susceptible to secondary dislocation, according to Liechti et al. [4]. Dunn et al. documented inferior functional outcomes for PLFDs relative to PLDs within the U.S. military population [9].

These injuries exhibit diverse and distinctive patterns of ligament and bone pathology in each case, yet they share the common feature of significant carpal bone misalignment. Due to the rarity of these conditions, large-scale studies are limited. This study aims to determine whether PLD and PLFD result in distinct radiological outcomes. We hypothesize that PLD and PLFD demonstrate differing radiological outcomes, shaped by variations in injury patterns and severity. Furthermore, this study seeks to provide a detailed analysis of the radiological features of these injuries—from diagnosis through surgical treatment and follow-up—while identifying radiological factors that may predict treatment outcomes.

## Material and method

The study was approved by the Ethics Committee of the University of Tübingen (project number 288/2021B02) in accordance with the ethical standards laid down in the Declaration of Helsinki. The study included all patients diagnosed with perilunate dislocations between January 2000 and December 2022, who underwent primary reconstructive wrist surgery and had a minimum of 12 months of radiological follow-up. To conduct the retrospective clinical analysis, the CGM database was queried using the ICD-10-CM code S63.0, which corresponds to “subluxation and dislocation of wrist and hand joints”. The initial cohort comprised 69 patients with perilunate injuries. Four patients were excluded due to undergoing primary treatment with proximal row carpectomy and 14 more were excluded due to insufficient follow-up duration. Ultimately, 51 of the 69 patients met the inclusion criteria, were of legal age, and were included in the study.

### Clinical data

A comprehensive review of the surgical records was conducted to obtain demographic and clinical data, including patient gender, age at the time of injury, the interval between injury and reduction (in days), the time from injury to surgical intervention (in days), duration of hospitalization (in days), the nature of the injury, the specific surgical procedures performed, and the presence of paresthesia upon admission.

### Radiological assessment

All radiographs were retrospectively stored and assessed using the digital Picture Archiving and Communication System (PACS) at our institution. A comprehensive analysis of the preoperative X-ray series in posteroanterior (PA) and lateral projections, as well as computed tomography, was conducted to ensure accurate injury classification. Moreover, intraoperative X-ray series in PA and lateral projections, along with follow-up radiographs in PA, lateral, and Stecher’s projections, were routinely reviewed.

### Radiological classification of the injury

**Fig. 1:**
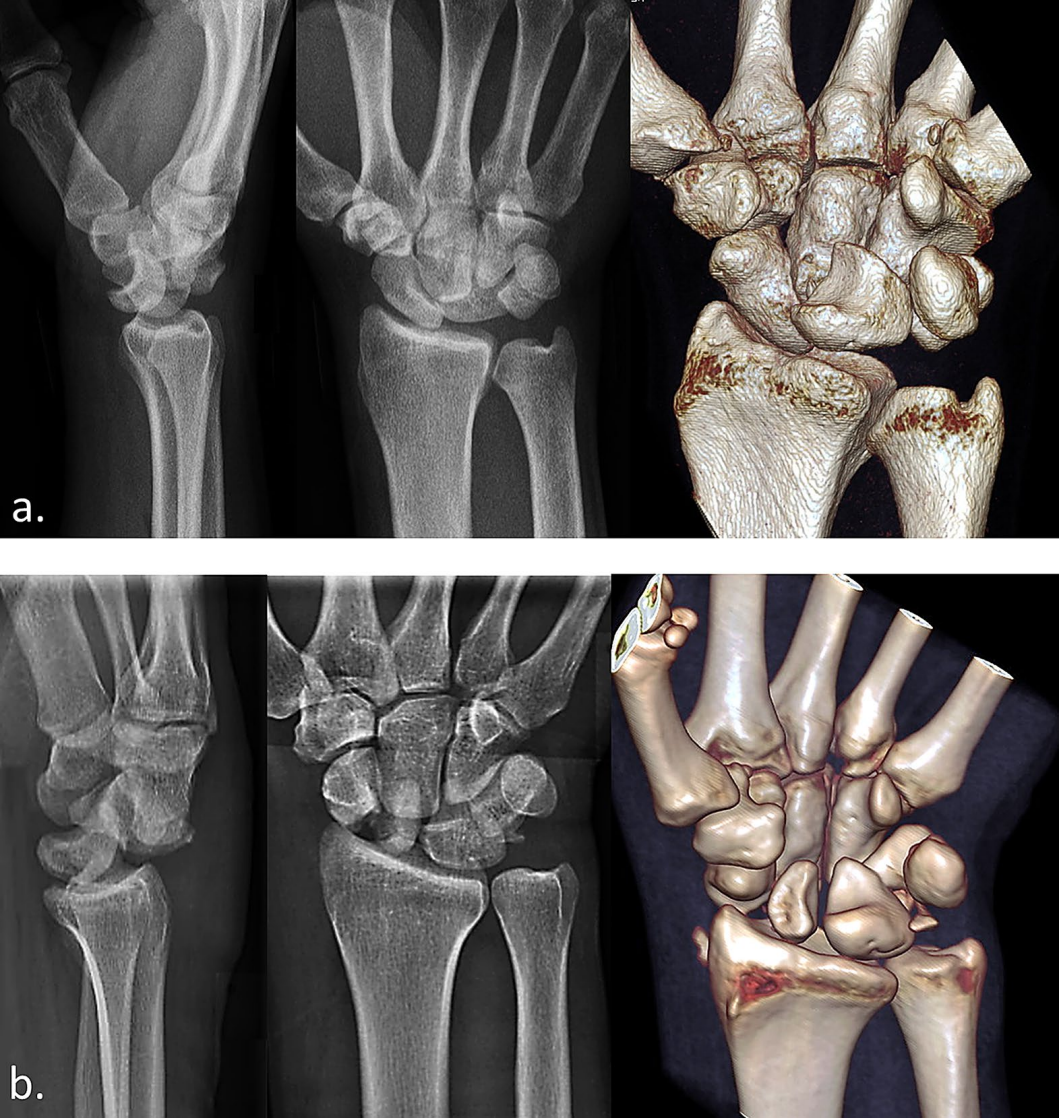
X-ray series in lateral and PA projections, along with corresponding CT scans with three-dimensional reconstructions of a ligamentous-only perilunate dislocation (**a**) and a trans-scaphoid perilunate fracture-dislocation (**b**)

Perilunate dislocations (PLD) were diagnosed in cases of ligamentous injuries without the presence of significant fractures (Fig. 1a). Perilunate fracture dislocations (PLFD) were identified when fractures of the wrist were present, with the exception of cases involving fractures of the radial or ulnar styloid tip, avulsion of the palmar triquetral insertion of the lunotriquetral interosseous ligament, or avulsion of the corticalis with involvement of the scapholunate ligament (Fig. 1b) [3]. Conversely, fractures that traverse the base of the radial styloid or the body of the triquetrum were classified as transradial styloid or transtriquetral PLFD variants. The stage of progressive perilunate instability was assessed using the Mayfield classification [10].

### Carpal alignment

According to the International Wrist Investigators’ Workshop guidelines, dorsal intercalated segment instability (DISI) was diagnosed in lateral X-ray projection with the wrist in neutral position. This was identified by a radial lunate angle (RLA) of -15° or less. In contrast, palmar intercalated segment instability (PISI) was diagnosed when the RLA was + 15° or greater [11].

### Scapholunate distance

To detect persisting postoperative scapholunate instability, the scapholunate-distance (SLD) was measured according to Schimmerl-Metz et al. at the most optimal anatomical site - at the midportion of the scapholunate joint in a PA and Stecher’s projection [12]. Pathological SLD was defined according to Dornberger et al. with a cut-off value of 3 mm for SLD in PA projection and/or of 3.7 mm for SLD in Stecher’s projection [13].

### Ulnar translocation

The Gilula method was used to analyze ulnar translocation. This involves measuring the length of the lunate bone extending beyond the lunate fovea, expressed as the ratio of|EF| (length beyond the fovea) to|EG| (width of the lunate bone). (|EF|)/(|EG|) values greater than 50% indicate ulnar translocation [14–16].

### Arthrosis

The severity of arthrosis after surgery was assessed using the Kellgren-Lawrence classification [17]. Arthrosis in the lunate fovea (FL), scaphoid fossa (FS), and midcarpal joint were assessed independently.

### Statistical method

In instances where the normal distribution was rejected, the Mann-Whitney-U test (MWU) was employed to compare two independent samples (for more than two groups, the Kruskal-Wallis test was applied). In the event that the Kruskal-Wallis test yielded statistically significant results, Dunn’s test with Bonferroni correction was conducted to precisely identify the specific groups that differed. Hence, the p-values presented from post-hoc tests were adjusted for multiple comparisons. To compare the frequency distributions of the categorical variables, the Fisher’s exact test was employed. In the study, both ordinal logistic regression and logistic regression models were used. The optimal form of the multivariable model was selected using a backward stepwise method, which minimizes the Akaike Information Criterion (AIC) [18]. The explanatory variables included in the analysis were: the type of injury (PLFD/PLD), Mayfield classification, capsulodesis, intercarpal ligament repair, repair of extrinsic ligaments and number of wrist transfixations. In the logistic regression analyses, the dependent variables considered were: pathological SLD (with responses “Yes” and “No”), ulnar translocation (with responses “Yes” and “No”), carpal alignment (“Normal” vs. “Abnormal” – with categories including PISI and DISI), lunate fovea arthrosis (assessed with responses “Normal” vs. “Other” – with categories ranging from 1 to 4), and scaphoid fossa arthrosis (assessed using the same scale and grouping). The impact of the explanatory variables on the dependent variable capitate/lunate midcarpal arthrosis [also assessed on a 0–4 scale but with group divisions: “Normal” (0) vs. “Doubtful + Mild” (1 + 2) vs. “Moderate + Severe” (3 + 4)] was evaluated using ordinal regression modeling. Spearman’s correlation coefficient was used to assess the strength and direction of monotonic relationships between both continuous and ordinal variables, particularly when the data did not meet the assumptions of normality or linearity. The level of significance was set to *p* ≤ 0.05, and the calculations were performed using R software (R Core Team (2022), version 4.3.2).

## Results

Table 1 provides a summary of the descriptive characteristics of the patients and their injuries.

**Table 1:** Clinical characteristics and injury details of patients with perilunate injuries

Variable	Parameter	Overall (*N* = 51)
Age at the time of the injury	N	51
	Mean (SD)	37.76 (15.26)
	Median (Q1-Q3)	37 (24–49)
	Range	18–72
Time from the injury to reposition [days]	N	51
	Mean (SD)	1.37 (2.81)
	Median (Q1-Q3)	0 (0–1)
	Range	0–15
Time from the injury to operation [days]	N	51
	Mean (SD)	2.18 (3.43)
	Median (Q1-Q3)	1 (0–2.5)
	Range	0–15
Hospitalization time [days]	N	51
	Mean (SD)	9.55 (9.21)
	Median (Q1-Q3)	6 (4–10.5)
	Range	2–52
Type of injury	PLD (Perilunate Dislocation)	45.1% (*N* = 23)
	PLFD (Perilunate Fracture Dislocation)	54.9% (*N* = 28)
Mayfield classification	Scapholunate dissociation (I)	0% (*N* = 0)
	I + Lunocapitate disruption (II)	29.4% (*N* = 15)
	II + Lunotriquetral disruption (III)	51% (*N* = 26)
	Lunate dislocation (IV)	19.6% (*N* = 10)
Scaphoid fracture	Yes	49% (*N* = 25)
	No	51% (*N* = 26)
Triquetrum fracture	Yes	21.6% (*N* = 11)
	No	78.4% (*N* = 40)
Processus styloideus radii fracture	Yes	29.4% (*N* = 15)
	No	70.6% (*N* = 36)
Processus styloideus ulnae fracture	Yes	9.8% (*N* = 5)
	No	90.2% (*N* = 46)
Number of fractures	N	51
	Mean (SD)	1.08 (0.89)
	Median (Q1-Q3)	1 (0–2)
	Range	0–4
Localization of scaphoid fracture	None	49% (*N* = 25)
	Waist	43.1% (*N* = 22)
	Proximal pole	5.9% (*N* = 3)
	Distal pole	2% (*N* = 1)
Intercarpal ligament injury	SL + LT	51% (*N* = 26)
	LT	21.6% (*N* = 11)
	SL	13.7% (*N* = 7)
	None	13.7% (*N* = 7)
Paresthesia upon admission	Yes	60.8% (*N* = 31)
	No	39.2% (*N* = 20)
Follow Up Time (Months)	N	51
	Mean (SD)	51.99 (58.18)
	Median (Q1-Q3)	31.2 (15.46–58.65)
	Range	12- 265.51

PLD: Perilunate dislocation PLFD: Perilunate fracture dislocation SL: Scapholunate ligament LT: Lunotriquetral ligament SD: Standard deviation Q1-Q3: Interquartile range (1st quartile − 3rd quartile)

The study analyzed 51 injury cases, with a mean patient age of approximately 37 years, ranging from 18 to 72 years of age. All patients were male. In 17 patients (33%), the injury was attributed to a fall from a ladder, roof, or balcony. In 15 patients (29%), the injury was sustained as a result of participation in sports activities, including soccer, motocross, mountain biking, snowboarding, or skiing. Fourteen patients (28%) were admitted to the hospital following a motor vehicle accident, including collisions involving cars, motorcycles, or bicycles. Three patients (6%) sustained injuries as a result of falls from standing height, while two patients (4%) suffered injuries due to workplace accidents involving machinery. Among the two injury types, PLFD was more prevalent, accounting for approximately 55% (*N* = 28) of cases. In accordance with the Mayfield classification system, no injuries were categorized as stage 1, while stage 3 constituted the majority (51%, *N* = 26) of cases. Scaphoid fractures were observed in nearly half (*N* = 25) of the patients, while triquetrum fractures occurred in approximately 22% (*N* = 11) of cases. Fractures of the radial styloid (PSR) and ulnar styloid (PSU) were observed in just under 30% (*N* = 15) and 10% (*N* = 5) of cases, respectively. The mean radiological follow-up period was 4.33 years, with a range of 12 months to 22 years.

The specifics of the surgical treatments and radiological outcomes are outlined in Table 2.

**Table 2:** Overview of performed surgical treatment and radiological indices in patients with perilunate injuries

Variable	Parameter	Overall (*N* = 51)
Surgical access	Both	51% (*N* = 26)
	Dorsal	47.1% (*N* = 24)
	Palmar	2% (*N* = 1)
Capsulodesis	Yes	27.5% (*N* = 14)
	None	72.5% (*N* = 37)
Ligament repair	Both (SL + LT)	43.1% (*N* = 22)
	SL	19.6% (*N* = 10)
	LT	17.6% (*N* = 9)
	None	19.6% (*N* = 10)
Ligament repair extrinsic ligaments	Dorsal	9.8% (*N* = 5)
	Dorsal + Palmar	7.8% (*N* = 4)
	Palmar	3.9% (*N* = 2)
	None	78.4% (*N* = 40)
Ligament repair extrinsic	Yes	21.6% (*N* = 11)
	No	78.4% (*N* = 40)
Number of wrist transfixations	N	51
	Mean (SD)	2.43 (0.94)
	Median (Q1-Q3)	3 (2–3)
	Range	0–4
Intraoperative/early postoperative reduction outcome	Excellent	74.5% (*N* = 38)
	Good	21.6% (*N* = 11)
	Fair	2% (*N* = 1)
	Poor	2% (*N* = 1)
	Very poor	0% (*N* = 0)
Pathologic SL Distance - intraoperative	Yes	7.8% (*N* = 4)
	No	92.2% (*N* = 47)
Carpal alignment - intraoperative	Normal	94.1% (*N* = 48)
	VISI	5.9% (*N* = 3)
	DISI	0% (*N* = 0)
Pathologic SL Distance at follow-up	Yes	17.6% (*N* = 9)
	No	82.4% (*N* = 42)
SL Distance (mm) at follow-up	N	51
	Mean (SD)	2.12 (1.19)
	Median (Q1-Q3)	1.85 (1.5–2.7)
	Range	0.5–5.3
RL angle (degrees) at follow-up	N	51
	Mean (SD)	10.13 (7.38)
	Median (Q1-Q3)	9.2 (3.6–14.4)
	Range	0–33
SL angle (degrees) at follow-up	N	51
	Mean (SD)	55.01 (10.44)
	Median (Q1-Q3)	56.5 (47.9–60)
	Range	30–77
Carpal alignment - at follow-up	Normal	72.5% (*N* = 37)
	DISI	19.6% (*N* = 10)
	VISI	7.8% (*N* = 4)
Ulnar translocation- at follow-up	Yes	25.5% (*N* = 13)
	No	74.5% (*N* = 38)
Lunate fossa arthrosis according to the Kellgren-Lawrence scale (0–4) at follow-up	Normal	72.5% (*N* = 37)
	Doubtful	15.7% (*N* = 8)
	Mild	7.8% (*N* = 4)
	Moderate	0% (*N* = 0)
	Severe	3.9% (*N* = 2)
Scaphoid fossa arthrosis according to the Kellgren-Lawrence scale (0–4) at follow-up	Normal	62.7% (*N* = 32)
	Doubtful	15.7% (*N* = 8)
	Mild	15.7% (*N* = 8)
	Moderate	2% (*N* = 1)
	Severe	3.9% (*N* = 2)
Capitate/lunate midcarpal arthrosis according to the Kellgren-Lawrence scale (0–4) at follow-up	Normal	41.2% (*N* = 21)
	Doubtful	13.7% (*N* = 7)
	Mild	17.6% (*N* = 9)
	Moderate	19.6% (*N* = 10)
	Severe	7.8% (*N* = 4)
Scaphoid nonunion	Yes	9.8% (*N* = 5)
	No	90.2% (*N* = 46)

DISI: Dorsal Intercalated Segment Instability

LT: Lunotriquetral ligament

Q1-Q3: Interquartile range (1st quartile − 3rd quartile)

SD: Standard deviation

SL: Scapholunate ligament

PISI: Palmar Intercalated Segment Instability

### Type of injury (PLD and PLFD)

**Fig. 2:**
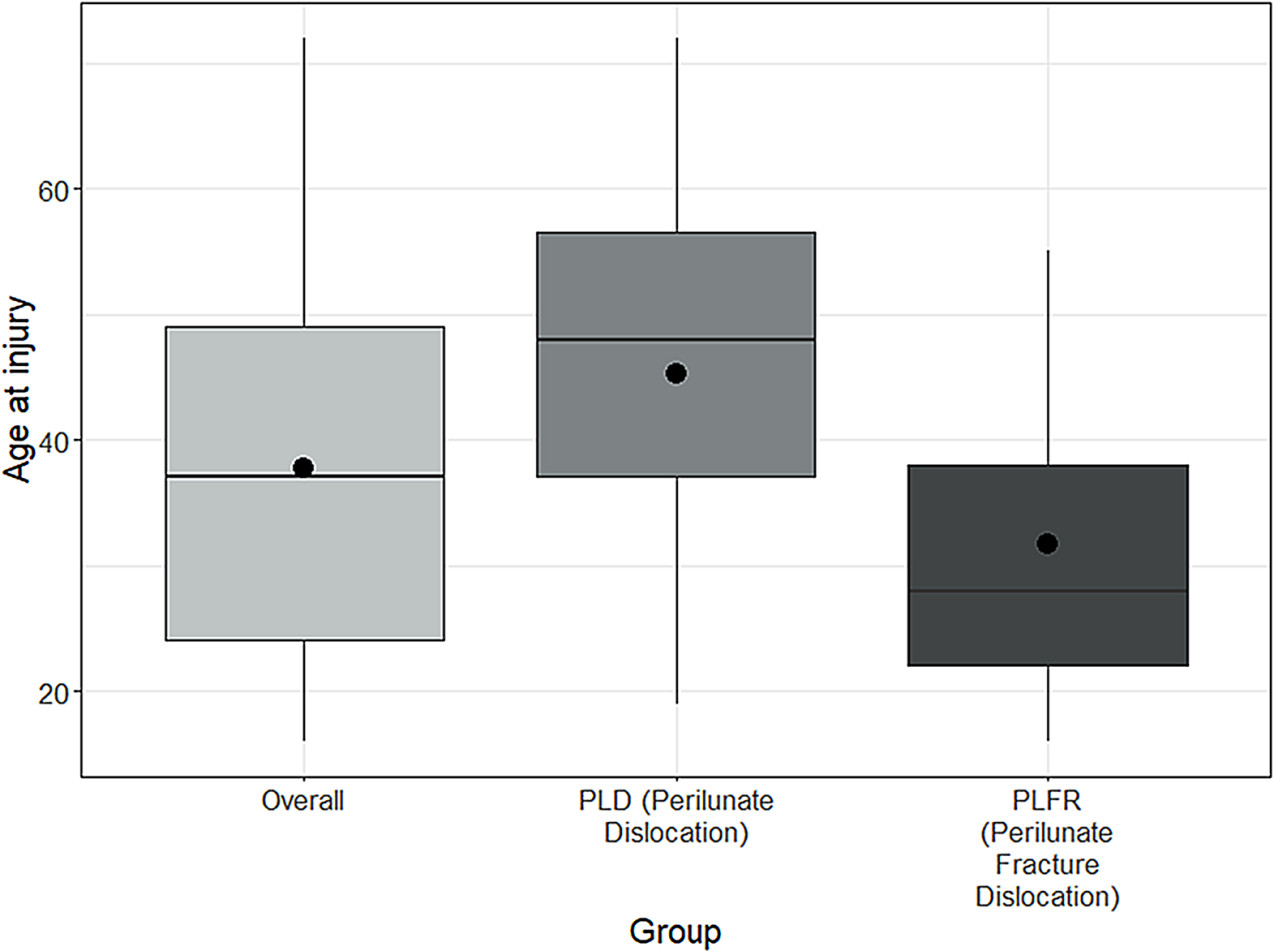
Box plots showing the differences in age between patients with perilunate dislocation (PLD) and perilunate fracture dislocation (PLFR)

The only significant clinical difference between the groups of patients with PLD and PLFD was found in age at the time of injury, with patients in the PLD group being significantly older. The median age was 48 years (Q1-Q3: 37–56.5) in the PLD group and 28 years (Q1-Q3: 22–38) in the PLFD group (Mann-Whitney U Test, *p* = 0.0031) (Fig. 2). No differences were observed with regard to the stage of perilunate instability, pathological SLD, ulnar translocation or paresthesia (*p* > 0.05). There was a slight tendency toward a higher number of transfixations in the case of PLD (median = 3), compared to PLFD (median = 2), which, however, did not reach statistical significance (*p* = 0.07).

### Stages of mayfield classification and reposition outcomes

**Fig. 3:**
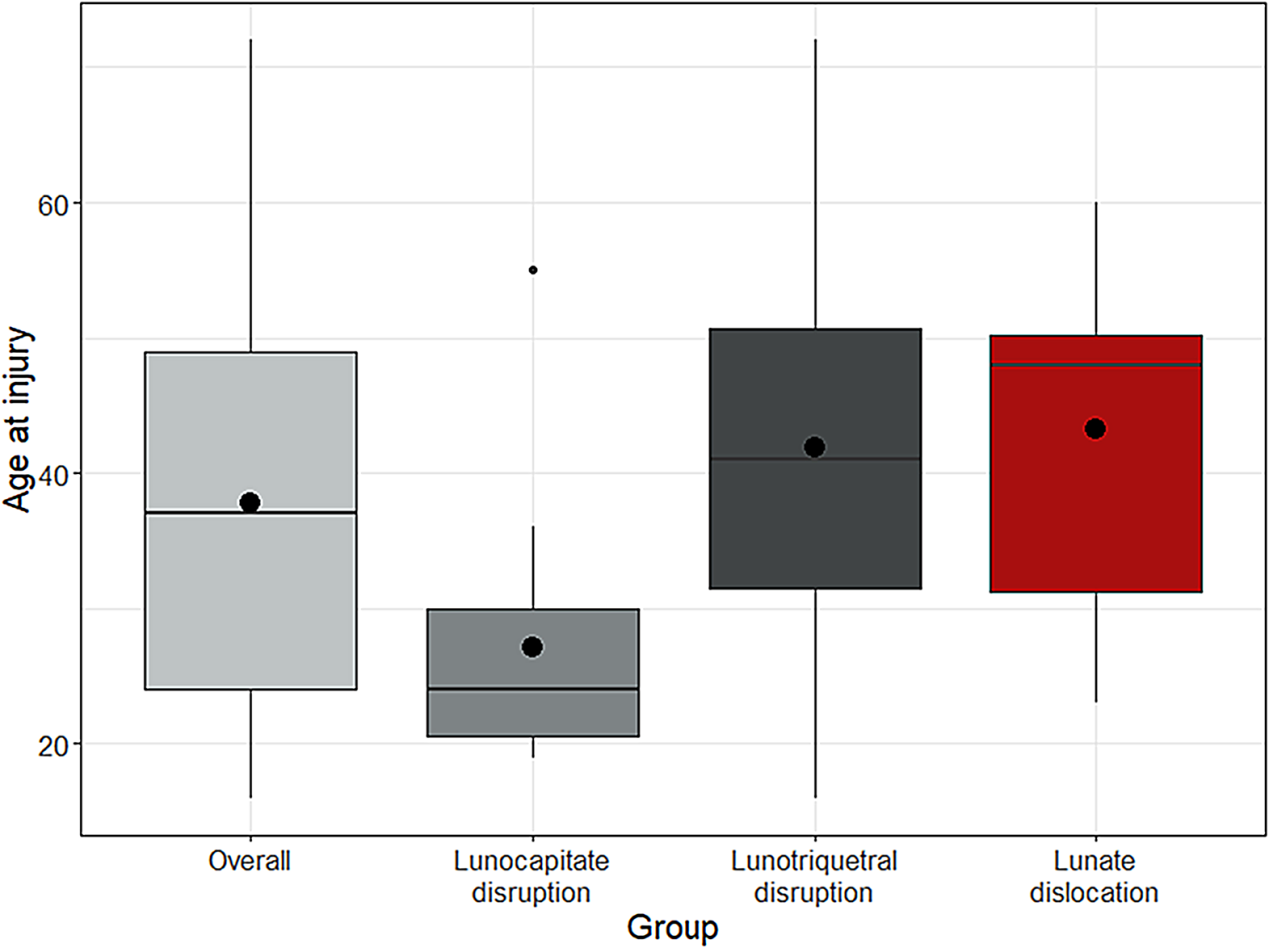
Box plots showing the differences in age between patients with different stages of perilunate instability according to the Mayfield classification (Stage I: Scapholunate dissociation, Stage II: additionally, lunocapitate disruption, Stage III: further lunotriquetral disruption, and Stage IV: Lunate dislocation)

The only significant difference between the Mayfield groups was found in the age of patients at the time of injury. The median age of patients with stage II perilunate instability was 24 (Q1-Q3: 20.5–30) comparing to 41 (31.5–50.75) in case of Mayfiled stage III and 48 (31.25–50.25) years of age in case of stage IV perilunate instability, respectively (Kruskal-Wallis, *p* = 0.0061). The post-hoc Dunn test revealed, that the differences were significant comparing the groups of patients with Stage IV and II of perilunate instability (*p* = 0.0113) and the groups of patients with stage II and III of Mayfield classification (*p* = 0.0063) (Fig. 3). Intraoperative reduction outcomes were assessed using a scale (0 = very poor, 1 = poor, 2 = fair, 3 = good, 4 = excellent) by a senior consultant. The number of wrist transfixations did not correlate with Mayfield Classification (*p* = 0.16), the number of fractures (*p* = 0.06), or intraoperative reduction outcome (*p* = 0.597).

### Carpal alignment at follow-up

Subsequent intergroup tests were conducted to identify the potential influence of the following variables: injury types (PLD, PLFD) and stages, intercarpal and extrinsic ligament reconstructions, capsulodesis, the number of wrist transfixations and the final carpal alignment at follow-up (classified as normal vs. abnormal, including DISI and PISI classifications). Nevertheless, no statistically significant differences were identified across any of the variables (*p* > 0.05). Furtherly, the logistic regression model used to predict carpal alignment was developed using the stepwise backward selection method, which starts with all potential predictors and progressively removes those that are least significant. The final model included only one predictor: capsulodesis. Capsulodesis demonstrated a trend toward improving carpal alignment, as suggested by the positive odds ratio of 2.72, indicating a potential association. However, despite being the only variable included in the model, capsulodesis was not identified as a statistically significant factor influencing carpal alignment (*p* = 0.136, OR = 2.72, LCI = 0.729, UCI = 10.1, McFadden’s pseudo-R²=0.037). Therefore, capsulodesis should not be universally applied as a necessary part of the standard therapy for perilunate luxation injuries.

### Ulnar translocation at follow-up

Ulnar translocation of the wrist was not associated with the type of injury (PLD/PLFD) (Fisher’s Exact Test, *p* = 0.207) or the stage of the Mayfield Classification (*p* = 0.405). Typically, repair of the extrinsic ligaments, primarily the palmar ligaments, is performed to prevent ulnar translocation of the wrist. In our study, however, we did not observe an influence of the repair of extrinsic ligaments on the development of ulnar translocation (Fisher’s Exact Test, *p* = 0.25). Therefore, we would not recommend it as a necessary step in the treatment of perilunate injuries.

### Analysis of wrist osteoarthrosis

#### Radiocarpal arthrosis in scaphoid fossa at follow-Up

To identify potential significant differences in scaphoid fossa condition, categorized as normal versus other (with degenerative changes according to Kellgren-Lawrence grades 1–4), intergroup tests were performed. Nevertheless, no statistically significant differences were identified between the groups with respect to any of the variables under consideration (*p* > 0.05).

#### Radiocarpal arthrosis in lunate fovea at follow-up

**Fig. 4:**
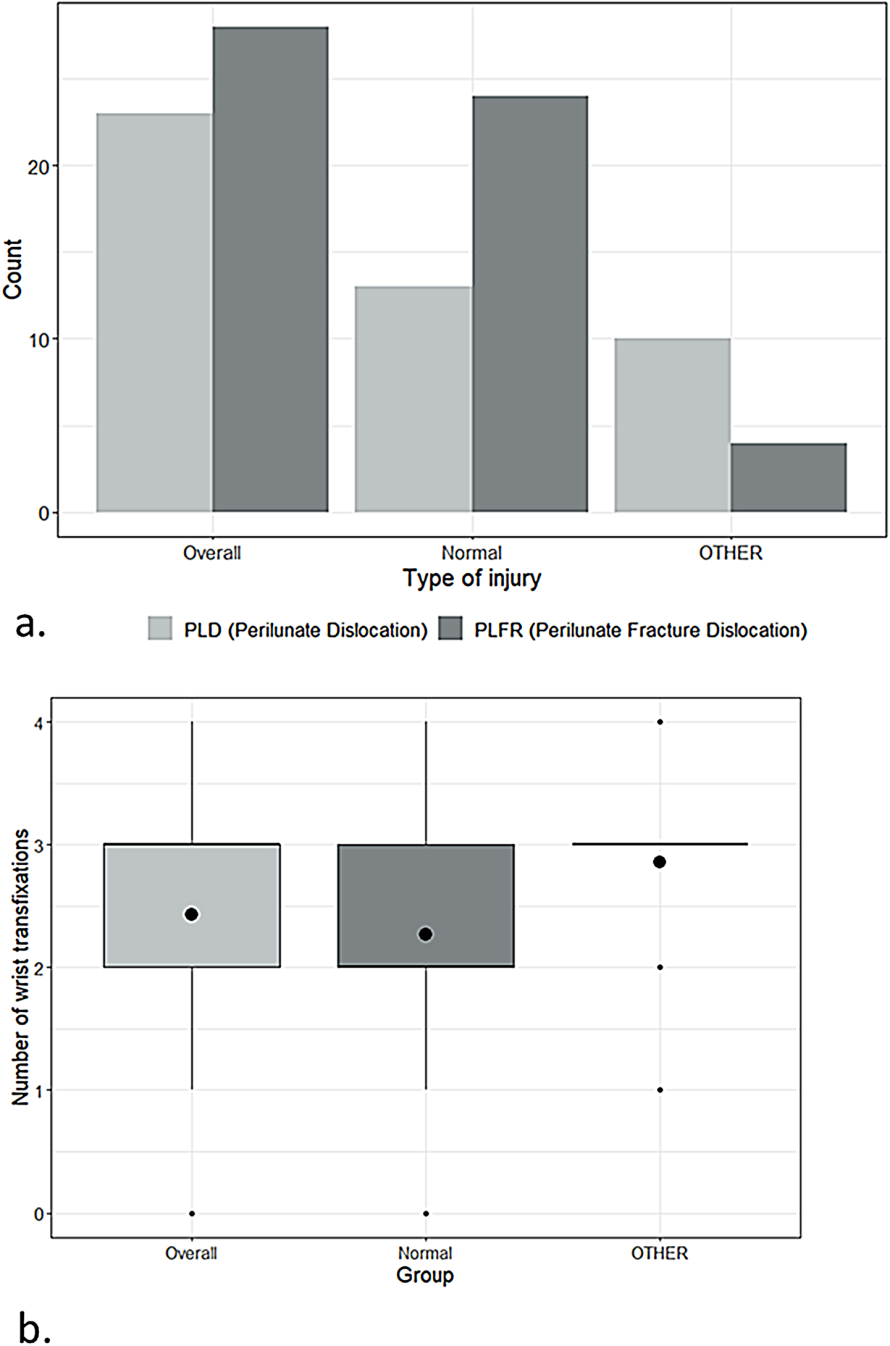
Graphs illustrating the relationship between arthrosis grades in the lunate fovea at follow-up and: (**a**) the type of injury, including perilunate dislocation (PLD) and perilunate fracture dislocation (PLFR), and (**b**) the grade of degenerative changes in relation to the number of wrist transfixations. The condition of the lunate fovea was classified as either normal or “other,” including stages 1–4 according to the Kellgren-Lawrence Classification

To assess the existence of notable discrepancies in lunate fovea condition, a categorization was employed wherein the normal group was contrasted with other categories, including those exhibiting degenerative alterations classified according to the Kellgren-Lawrence grading system (grades 1–4). To this end, intergroup tests were conducted, incorporating the aforementioned variables. Two variables demonstrated significant statistical differences between the groups. First, the type of injury varied significantly, with a higher prevalence of PLFD (64.9%, *N* = 24 vs. for PLD: 35.1%, *N* = 13) in the group without arthrosis, while PLD was more common in the group with apparent degeneration [71.4%, *N* = 10 vs. for PLFD: 28.6%, *N* = 4 (Fisher’s exact test, *p* = 0.0286)] (Fig. 4a). Secondly, the number of wrist transfixations was significantly lower in the group without signs of lunate fovea arthrosis (median = 2, Q1-Q3 = 2–3, *N* = 37) compared to those with arthrosis [median = 3, Q1-Q3 = 3–3, *N* = 14 (Mann-Whitney U Test, *p* = 0.0382)] (Fig. 4b).

The logistic regression model revealed a positive correlation between the number of wrist transfixations and lunate fossa arthrosis (p-value = 0.048) (Table 3). However, the confidence interval for the odds ratio indicates a high level of uncertainty in its estimation. The Nagelkerke’s pseudo-R^2^ indicate a modest fit to the data, explaining only approximately 32.1% of the variance.

**Table 3:** The results of the logistic regression multivariate model for lunate fovea arthrosis include explanatory variables selected based on AIC (Akaike Information Criterion) values using stepwise forward selection. The regression coefficients (Estimate) indicate the direction and strength of the relationship between each variable and lunate fovea arthrosis. Negative estimates suggest a protective effect, while positive estimates suggest increased odds. The odds ratio (OR) quantifies the change in odds associated with each variable. For “Number of wrist transfixations,” the OR is 3.529, suggesting a 3.5-fold increase in odds per additional transfixation. The confidence intervals provide a range of plausible values for the OR (LCI-UCI). If the LCI is below 1 and the UCI is above 1, the result is not statistically significant, as it includes the possibility of no effect (OR = 1). McFadden’s pseudo-R2 and Nagelkerke’s pseudo-R2 assess how well the model explains the variability in the outcome, with values closer to 1 indicating a better fit

Variable	Estimate	*p*-value	OR	LCI	UCI
Ligament repair: SL	-0.811	0.535	0.444	0.034	5.78
Ligament repair: LT	-18.899	0.992	0.000	0.000	Inf
Ligament repair: Both (SL + LT)	-0.642	0.598	0.526	0.048	5.72
Number of wrist transfixations	1.261	0.05*	3.529	1.002	12.43
*Indicators of model fit*					
**McFadden**	**Nagelkerke**		**AIC**		**BIC**
0.213	0.321		57.2		66.8

AIC: Akaike Information Criterion BIC: Bayesian Information Criterion LCI: Lower Confidence Interval LT: Lunotriquetral ligament OR: Odds Ratio SL: Scapholunate ligament UCI: Upper Confidence Interval

Additionally, arthrosis in the lunate fovea correlated weakly with the intraoperative RL angle (p-value = 0.035, Spearman’s rho = 0.296.

#### Midcarpal arthrosis between capitate und lunate at follow-up

At the follow-up examination, midcarpal arthrosis was the most prevalent form of arthritis observed in the studied population, with only 41.2% (*N* = 21) showing no signs of midcarpal degeneration. In 13 out of 23 cases (57%) of diagnosed osteoarthritis (Grade 2 or higher), the pathology was localized solely to the joint space between the capitate and lunate. Due to the larger sample sizes available for comparison, a more detailed analysis of degenerative changes was conducted using groups classified as 0, 1–2, and 3–4 according to the Kellgren-Lawrence Classification, as this classification system allows for a more nuanced understanding of the observed phenomena. As with the findings in lunate fovea arthrosis, two variables demonstrated significant statistical differences between groups. First, the type of injury (PLD/PLFD) was found to be significantly associated with arthrosis grades, with a greater proportion of patients with PLD in the group with more advanced arthrosis (Fischer’s exact test, *p* = 0.0071) (Table 4). Secondly, an increased number of wrist transfixations was found to be correlated with more severe midcarpal joint degeneration (Kruskal-Wallis, *p* = 0.0156) (Table 4). The Dunn post-hoc test revealed statistically significant differences between the 3–4 grade midcarpal arthrosis group and the group with no degenerative changes (*p* = 0.006) with regard to the number of wrist transfixations.

**Table 4:** Intergroup comparisons of capitate/lunate midcarpal arthrosis by injury types—Perilunate dislocation (PLD) and Perilunate Fracture dislocation (PLFR)—and the number of wrist transfixations. The radiological condition of the lunate fovea was classified as 0 (normal), 1–2 (doubtful + mild arthrosis), and 3–4 (moderate + severe degenerative changes) according to the Kellgren-Lawrence scale. Furthermore, the results of the ordinal regression multivariate model, including explanatory variables chosen based on AIC values using stepwise forward selection, are shown. The third section of the table includes the statistical indicators of model fit

Variable	Parameter	Overall (*N* = 51)	Normal (*N* = 21)	Doubtful + Mild (*N* = 16)	Moderate + Severe (*N* = 14)	test	*p*-value
Type of injury	PLD (Perilunate Dislocation)	45.1% (*N* = 23)	19% (*N* = 4)	62.5% (*N* = 10)	64.3% (*N* = 9)	Fisher	0.0071*
	PLFR (Perilunate Fracture Dislocation)	54.9% (*N* = 28)	81% (*N* = 17)	37.5% (*N* = 6)	35.7% (*N* = 5)		
Number of wrist transfixations	N	51	21	16	14	Kruskal-Wallis	0.0156*
	Mean (SD)	2.43 (0.94)	2 (1.1)	2.56 (0.63)	2.93 (0.73)		
	Median (Q1-Q3)	3 (2–3)	2 (1–3)	2.5 (2–3)	3 (3–3)		
	Range	0–4	0–4	2–4	1–4		
Capitate/lunate midcarpal arthrosis– ordinal regression multivariate model							
**Variable**	**Estimate**		**OR**		**LCI**	**UCI**	**p-value**
Type of injury - PLFR (Perilunate Fracture Dislocation)	-1.227		0.293		0.094	0.917	0.04*
Number of wrist transfixations	0.887		2.427		1.162	5.068	0.022*
Indicators of model fit							
**McFadden**	**Nagelkerke**		**AIC**		**BIC**		
0.136	0.289		104		111		

AIC: Akaike Information Criterion BIC: Bayesian Information Criterion LCI: Lower Confidence Interval LT: Lunotriquetral ligament OR: Odds Ratio SL: Scapholunate ligament UCI: Upper Confidence Interval

According to the multivariate ordinal regression model, the estimate for PLFD is -1.227, suggesting that patients with PLFD injuries have a lower risk of developing capitate-lunate midcarpal arthrosis compared to those with PLD injuries. The odds ratio (OR) reached 0.293, indicating that the odds of developing a more severe category of arthrosis are reduced by 70.7% in patients with PLFD injuries compared to patients with PLD injuries. The confidence interval for the OR achieved 0.094 to 0.917, demonstrating a significant statistical difference, confirmed by a p-value of 0.04 (Table 4).

Furthermore, the estimate of 0.887 indicates that a higher number of wrist transfixations is associated with an increased risk of developing more advanced midcarpal joint arthrosis. The OR is equal to 2.427, indicating that the risk of progression to a higher category of arthrosis is more than double that associated with a single transfixation. The confidence interval was 1.162 to 5.068, with a p-value of 0.022, thereby confirming that this association is statistically significant. The McFadden’s pseudo-R² was 0.136 and the Nagelkerke’s pseudo-R^2^ reached a value of 0.289, indicating a moderate relationship (Table 4).

**Fig. 5:**
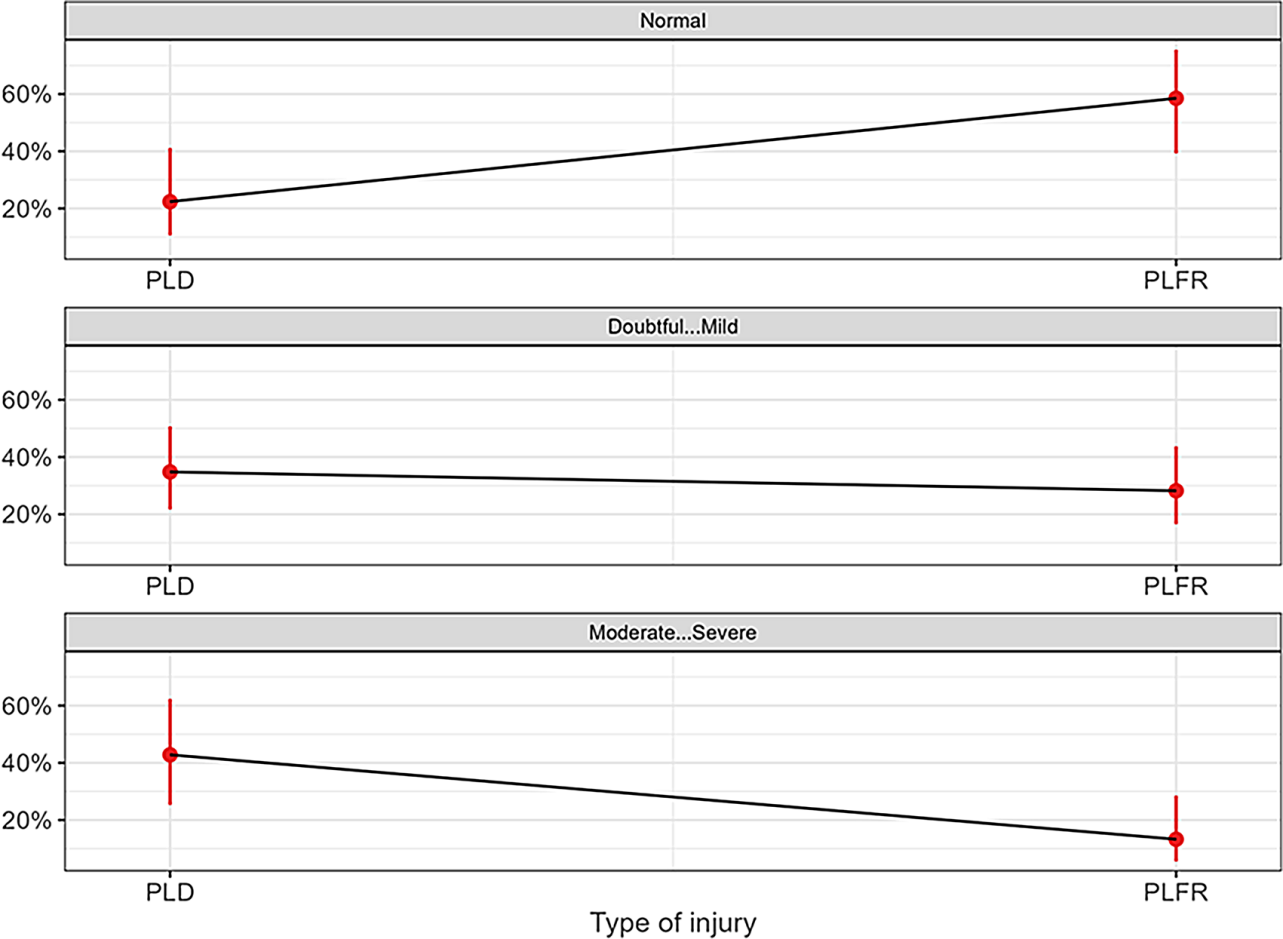
Dependency between capitate/lunate midcarpal arthrosis and type of injury. The relationship between capitate/lunate midcarpal arthrosis, classified according to the Kellgren-Lawrence scale, and type of injury (perilunate dislocation [PLD] or perilunate fracture-dislocation [PLFD]) are shown. The Kellgren-Lawrence scale groups were categorized into three levels: 0 (normal), 1–2 (doubtful to mild), and 3–4 (moderate to severe) arthrosis grades

**Fig. 6:**
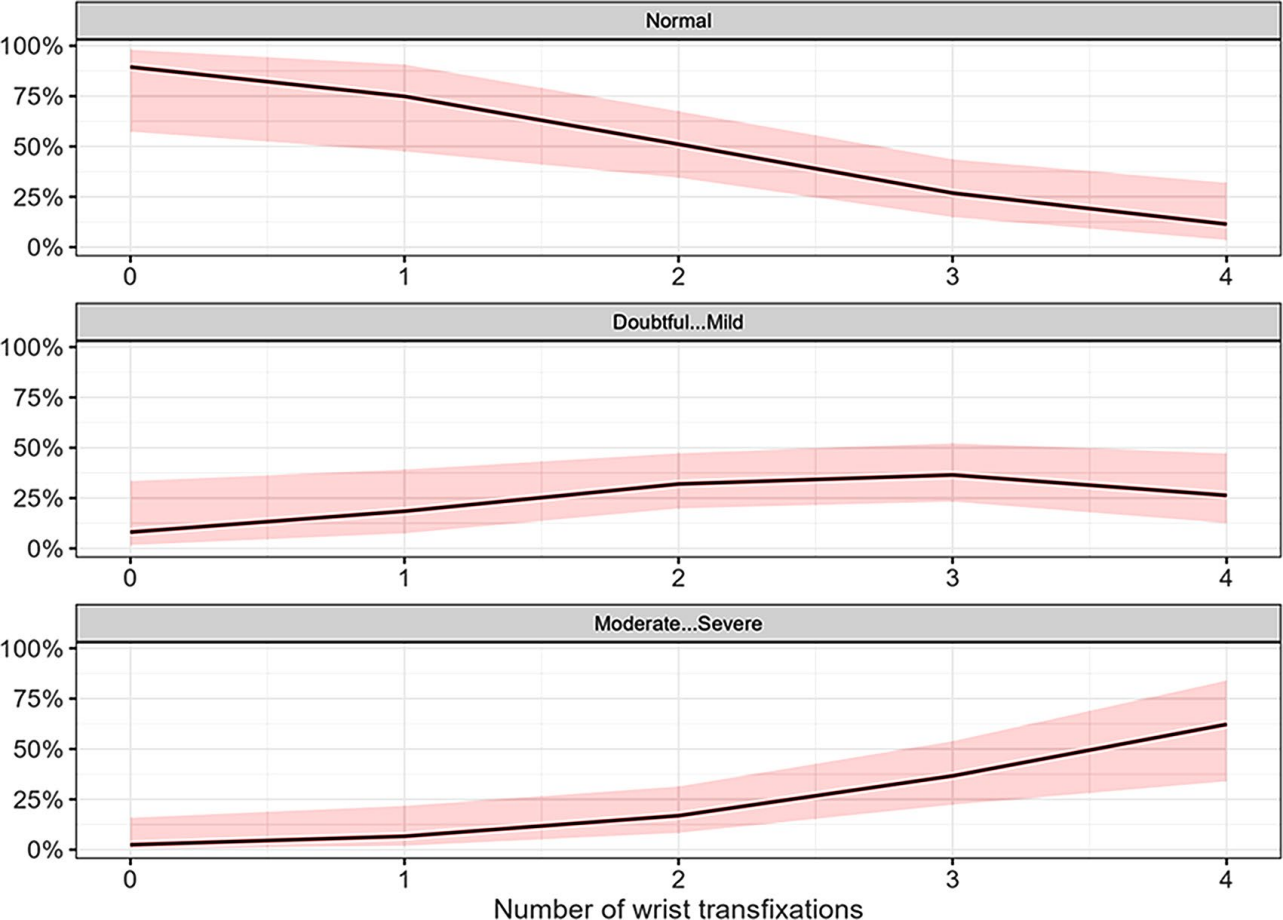
Dependency between capitate/lunate midcarpal arthrosis and the number of wrist transfixations. The arthrosis grades were categorized into three levels: 0 (normal), 1–2 (doubtful to mild), and 3–4 (moderate to severe)

In summary, patients with PLD injuries have an estimated probability of about 20% for a favorable outcome without arthritis, whereas patients with PLFD injuries have approximately a 60% probability of maintaining normal chondral conditions in the midcarpal joint. The probability of moderate to severe degenerative changes in the midcarpal joint is 40% for PLD and only 15% for PLFD (Fig. 5). Furthermore, as the number of wrist transfixations increases, the chance of a normal outcome decreases, while the risk of a more severe arthrosis outcome increases. The probability of a normal outcome is highest in cases where up to two transfixations have been performed. However, with three transfixations, the chances of doubtful/mild and moderate/severe arthrosis become nearly equal. From four transfixations onwards, moderate to severe arthrosis becomes the most likely outcome (Fig. 6).

Moreover, correlation analysis revealed that the intraoperatively assessed reduction outcome correlated moderately with midcarpal arthrosis at follow-up (*p* = 0.037, Spearman’s rho = -0.292).

## Discussion

In this study, perilunate fracture dislocation (PLFD) was diagnosed in 54.9% (*N* = 28) of cases. Similar to the meta-analysis conducted by Kussmaul et al., we observed that scaphoid fractures, followed by triquetrum fractures, are the most common bony injuries associated with perilunate luxations [19]. Consistent with findings from other authors, perilunate fracture dislocations are reported to be more prevalent than perilunate dislocations. For instance, Herzberg et al. observed a PLFD rate of 66%, Forli et al. reported 61%, Israel et al. 72%, Dunn et al. 55%, and Garçon et al. 78% [2, 3, 8, 9, 20, 21].

We observed a higher incidence of perilunate dislocations and more severe wrist derangements, according to the Mayfield classification, in older patients. To the best of our knowledge, this observation has not been previously reported in the literature. In vivo animal studies have demonstrated age-related changes in ligament properties, including increased failure strain and reduced Lubricin/PRG4 gene expression [22]. Additionally, in older adults, a progressive increase in collagen fibers and a decrease in oxytalan elastic fibers reduce ligament elasticity of the cervical interspinous ligament [23]. Scaphoid fractures are also significantly less common in older patients, with an incidence of 2.34 per 100,000 per year in individuals aged 20–29, compared to 0.5 per 100,000 per year in those aged 60–69 [24]. The higher incidence of PLD and more advanced stages of perilunate instability observed with age may be explained by age-related changes in ligament properties, a possible history of previous traumas, and degenerative changes in the intercarpal ligaments.

The most common complication of perilunate in injuries is wrist osteoarthritis, which occurs in 40–56% of patients, as reported in comparable follow-up studies [4, 25, 26]. Herzberg et al. identified open injuries and delayed treatment as significant risk factors for poorer clinical outcomes. In the same study, they found that 87% of perilunate dislocations and 69% of perilunate fracture-dislocations showed signs of arthritis at follow-up [3]. A recent systematic review by Liechti et al. also associated perilunate dislocations with a higher complication rate and an increased incidence of postoperative reduction loss (24% vs. 7%) [4]. These findings align with our observation that PLD might be associated with worse radiological outcome and a higher rate of wrist osteoarthritis. However, this may also be partially attributed to the increased susceptibility of older patients to ligament injuries, as indicated in our study.

Longer follow-up periods have been associated with higher rates of wrist degenerative changes. However, the majority of studies have found no significant correlation between these radiological changes and functional outcomes [2, 4, 8, 27, 28]. In comparison to our cohort, studies conducted in a younger population (mean age 28.8 years) suggest that patients with PLD may experience superior clinical outcomes, including reduced pain and a higher frequency of return to sports activities, in comparison to those with PLFD [9]. Conversely, Laporte et al. reported better functional outcomes in patients with PLFD at an average follow-up of 26 months [29]. Garçon et al. found that patients with large lunate displacements (Stage IIB) had lower DASH scores, indicating better outcomes [21]. Massoud et al. reported that, among patients with chronic perilunate luxations, approximately 70% of those with greater arc injuries and 33% of those with lesser arc lesions achieved good to excellent outcomes, as measured by the Mayo wrist score [30]. These varying results underscore the need for further functional studies comparing perilunate dislocations and perilunate fracture-dislocations in terms of clinical outcomes, while also contextualizing them with radiological findings.

The current literature lacks consensus regarding the optimal method for carpal transfixation in perilunate dislocations. Souer et al. demonstrated that temporary headless compression screws at the SL and lunotriquetral LT joints yielded results comparable to traditional K-wire transfixation, with the potential for shorter splinting durations in the future when using screws [31]. Song et al. advocate for addressing ulnar translocation of the wrist more extensively by using temporary radiolunate transfixation, as they reported a significantly higher rate of ulnar translocation, reaching up to 80% following perilunate injuries [15]. Furthermore, addressing this complication through pinning during the initial treatment resulted in improvements in the ulnar translocation index. In our study, the incidence of ulnar translocation of the carpus was 25.5%, with radiolunate transfixation performed in one case. This suggests that this technique may be necessary in selected cases.

Özyürekoğlu et al. reported favorable clinical outcomes but only moderate radiological results following the use of four K-wires in a diamond-shaped configuration to stabilize the SL, LT scaphocapitate, and triquetrohamate joints [32]. This was based on a relatively short mean follow-up period of 27 months (range 13–74 months), with an observed arthrosis rate of approximately 40%. Unexpectedly, our findings indicate that a greater number of wrist transfixations — particularly four— may be associated with an increased risk of radiocarpal and midcarpal arthrosis. A review of the existing literature reveals no similar observations, and this factor has generally not been subjected to direct analysis. A number of explanations could account for this phenomenon, including the potential for additional chondral damage during K-wire placement. Alternatively, the need for a higher number of transfixations may indicate a more severe underlying wrist derangement. As we did not observe a correlation between the stages of the Mayfield classification or between reduction outcomes and the number of wrist transfixations, we recommend restricting wrist pinning to essential procedures only, typically limiting it to a maximum of three transfixations, including those of the SL, LT, and scaphocapitate joints. The reconstruction of normal wrist alignment should always be a priority to reduce the risk of midcarpal and radiocarpal arthrosis.

The study is limited by its retrospective design, the small patient sample resulting from the low incidence of perilunate dislocations, and the heterogeneous nature of the study population with respect to injury types. Furthermore, the heterogeneity of treatment options based on the unique experiences and preferences of the treating surgeons introduces complexity in analyzing correlations between various factors and outcomes. Another limitation is the relatively brief period of radiological follow-up period, with a minimum of one year in some cases. Moreover, this study focuses on radiological outcomes, leaving room for future research to explore how these findings relate to broader aspects of wrist function and patient-reported outcomes.

## Data Availability

No datasets were generated or analysed during the current study.

## References

[1] Minami A, Kaneda K (1993) Repair and/or reconstruction of scapholunate interosseous ligament in lunate and perilunate dislocations. J Hand Surg Am 18:1099–1106. 10.1016/0363-5023(93)90410-5 8294749 10.1016/0363-5023(93)90410-5

[2] Herzberg G, Forissier D (2002) Acute dorsal trans-scaphoid perilunate fracture-dislocations: medium-term results. J Hand Surg Br 27:498–502. 10.1054/jhsb.2002.0774 12475503 10.1054/jhsb.2002.0774

[3] Herzberg G, Comtet JJ, Linscheid RL et al (1993) Perilunate dislocations and fracture-dislocations: a multicenter study. J Hand Surg Am. 10.1016/0363-5023(93)90041-Z.18:768– 79 8228045 10.1016/0363-5023(93)90041-Z

[4] Liechti R, Merky DN, Grobbelaar AO et al (2023) Outcomes of acute perilunate injuries—a systematic review. Eur J Trauma Emerg Surg 49(5):2071–2084. 10.1007/s00068-023-02222-y 36750472 10.1007/s00068-023-02222-y

[5] van der Oest MJW, Duraku LS, Artan M et al (2022) Perilunate Injury timing and treatment options: a systematic review. J Wrist Surg 11:164–176. 10.1055/s-0041-1735841 35478950 10.1055/s-0041-1735841 PMC9038303

[6] Soejima O, Iida H, Naito M (2003) Transscaphoid-transtriquetral perilunate fracture dislocation: report of a case and review of the literature. Arch Orthop Trauma Surg 123:305–307. 10.1007/s00402-003-0521-0 12783243 10.1007/s00402-003-0521-0

[7] Lee CH, Lee BG, Kim JH et al (2023) Complications and outcomes of operative treatment for acute perilunate injuries: a systematic review. J Hand Surg Eur 48:625–629. 10.1177/17531934221150331 10.1177/17531934221150331 36708152

[8] Forli A, Courvoisier A, Wimsey S et al (2010) Perilunate dislocations and Transscaphoid Perilunate Fracture-Dislocations: a retrospective study with Minimum Ten-Year Follow-Up. J Hand Surg Am 35:62–68. 10.1016/j.jhsa.2009.09.003 19931988 10.1016/j.jhsa.2009.09.003

[9] Dunn J, Koehler L, Kusnezov N et al (2018) Perilunate dislocations and Perilunate fracture dislocations in the U.S. military. J Wrist Surg 07:057–065. 10.1055/s-0037-1603932 10.1055/s-0037-1603932 PMC5788754 29383277

[10] Mayfield JK (1980) Mechanism of carpal injuries. Clin Orthop Relat Res 149:45. 10.1097/00003086-198006000-00006 7408319

[11] Gilula LA, Mann FA, Dobyns JH, Yin Y (2002) Wrist terminology as defined by the international wrist investigators’ workshop (IWIW). JBJS 84(suppl1):S1–S66

[12] Schimmerl-Metz SM, Metz VM, Totterman SMS et al (1999) Radiologic measurement of the scapholunate joint: implications of biologic variation in scapholunate joint morphology. J Hand Surg Am 24:1237–1244. 10.1053/jhsu.1999.1237 10584947 10.1053/jhsu.1999.1237

[13] Dornberger JE, Rademacher G, Mutze S et al (2015) Accuracy of simple plain radiographic signs and measures to diagnose acute scapholunate ligament injuries of the wrist. Eur Radiol 25:3488–3498. 10.1007/s00330-015-3776-2 25981221 10.1007/s00330-015-3776-2

[14] Berschback JC, Kalainov DM, Husain SN et al (2012) Traumatic ulnar translocation of the carpus: early recognition and treatment. J Hand Surg Eur 37:755–764. 10.1177/1753193412436626 10.1177/1753193412436626 22357328

[15] Song D, Goodman S, Gilula LA, Wollstein R (2009) Ulnocarpal translation in perilunate dislocations. J Hand Surg Eur Vol 34:38890. 10.1177/1753193409103093 10.1177/1753193409103093 19457905

[16] Gilula LA, Weeks PM (1978) Post-traumatic ligamentous instabilities of the wrist. Radiology.129:641– 51. 10.1148/129.3.641 10.1148/129.3.641 725039

[17] Kellgren JH, Lawrence JS (1957) Radiological assessment of osteo-arthrosis. Ann Rheum Dis 16:494–502. 10.1136/ard.16.4.494 13498604 10.1136/ard.16.4.494 PMC1006995

[18] Akaike H (1998) Information theory and an extension of the maximum likelihood principle. Selected papers of Hirotugu Akaike. Springer New York, New York, NY, pp 199–213

[19] Kussmaul AC, Kuehlein T, Langer MF, Ayache A, Löw S, Unglaub F (2024) The Conservative and Operative Treatment of Carpal Fractures. Dtsch Arztebl Int. 6:arztebl.m2024.0102. 10.3238/arztebl.m2024.0102 10.3238/arztebl.m2024.0102 PMC11661489 38863274

[20] Israel D, Delclaux S, André A et al (2016) Peri-lunate dislocation and fracture-dislocation of the wrist: retrospective evaluation of 65 cases. Orthop Traumatol Surg Res 102:351–355. 10.1016/j.otsr.2016.01.004 26897257 10.1016/j.otsr.2016.01.004

[21] Garçon C, Degeorge B, Coulet B et al (2022) Perilunate dislocation and fracture dislocation of the wrist: outcomes and long-term prognostic factors. Orthop Traumatol Surg Res 108:103332. 10.1016/j.otsr.2022.103332 35609818 10.1016/j.otsr.2022.103332

[22] Thornton GM, Lemmex DB, Ono Y et al (2015) Aging affects mechanical properties and lubricin/PRG4 gene expression in normal ligaments. J Biomech 48:3306–3311. 10.1016/j.jbiomech.2015.06.005 26163751 10.1016/j.jbiomech.2015.06.005

[23] Barros EMKP, Rodrigues CJ, Rodrigues NR et al (2002) Aging of the elastic and collagen fibers in the human cervical interspinous ligaments. Spine J 2:57–62. 10.1016/S1529-9430(01)00167-X 14588289 10.1016/s1529-9430(01)00167-x

[24] Van Tassel DC, Owens BD, Wolf JM (2010) Incidence estimates and demographics of scaphoid fracture in the U.S. population. J Hand Surg Am 35:1242–1245. 10.1016/j.jhsa.2010.05.017 20684922 10.1016/j.jhsa.2010.05.017

[25] Abola MV, Gerber BA, Rocks MC et al (2024) A comparison of outcomes in Acute Perilunate injuries: systematic review and Meta-analysis of treatment approaches. Hand. (N Y).15589447241231291 10.1177/15589447241231291 10.1177/15589447241231291 PMC11571428 38415721

[26] Pappa E, Argyrou C, Tetsios G et al (2024) Surgical management and functional outcomes of perilunate dislocations and fracture dislocations through the dorsal approach. Eur J Orthop Surg Traumatol 34:2751–2756. 10.1007/s00590-024-03999-3 38761199 10.1007/s00590-024-03999-3

[27] Kremer T, Wendt M, Riedel K et al (2010) Open reduction for Perilunate injuries-clinical outcome and patient satisfaction. J Hand Surg Am. 10.1016/j.jhsa.2010.06.021. 35:1599– 606 20888496 10.1016/j.jhsa.2010.06.021

[28] Meszaros T, Vögelin E, Mathys L, Leclère FM (2018) Perilunate fracture-dislocations: clinical and radiological results of 21 cases. Arch Orthop Trauma Surg 138:287–297. 10.1007/s00402-017-2861-1 29282524 10.1007/s00402-017-2861-1

[29] Laporte M, Michot A, Choughri H et al (2012) Perilunate dislocations and fracture-dislocations of the wrist, a review of 17 cases. Chir Main 31:6270. 10.1016/j.main.2012.01.010 10.1016/j.main.2012.01.010 22364827

[30] Massoud AHA, Naam NH (2012) Functional outcome of open reduction of chronic perilunate injuries. J Hand Surg Am 37:1852–1860. 10.1016/j.jhsa.2012.06.009 22854256 10.1016/j.jhsa.2012.06.009

[31] Souer JS, Rutgers M, Andermahr J, Jupiter JB, Ring D (2007) Perilunate fracture-dislocations of the wrist: comparison of temporary screw versus K-wire fixation. J Hand Surg Am 32:318–325. 10.1016/j.jhsa.2007.01.008 17336837 10.1016/j.jhsa.2007.01.008

[32] Özyürekoğlu T, Acar MA (2021) Treatment of acute perilunate dislocation or fracture dislocation using dorsal approach and diamond-shaped Kirschner-Wire fixation. Jt Dis Relat Surg 32:42–50. 10.5606/ehc.2021.74838 33463417 10.5606/ehc.2021.74838 PMC8073432

